# *Schizophyllum commune* Exopolysaccharides Reduce *Salmonella* Gut Epithelial Invasion and Activate Macrophages Towards M1-Polarization

**DOI:** 10.3390/ijms27104476

**Published:** 2026-05-16

**Authors:** Arishabhas Tantibhadrasapa, Pattarapon Boonpan, Thanawut Chotmanee, Songphon Buddhasiri, Jaturong Kumla, Nakarin Suwannarach, Saisamorn Lumyong, Parameth Thiennimitr

**Affiliations:** 1 Department of Microbiology, Faculty of Medicine, Chiang Mai University, Chiang Mai 50200, Thailand; arishabhas.t@gmail.com (A.T.);; 2 Department of Biology, Faculty of Science, Chiang Mai University, Chiang Mai 50200, Thailandjaturong.kumla@cmu.ac.th (J.K.); nakarin.su@cmu.ac.th (N.S.);; 3 Veterinary Public Health and Food Safety Centre for Asia Pacific, Faculty of Veterinary Medicine, Chiang Mai University, Chiang Mai 50100, Thailand; 4 Faculty of Veterinary Medicine, Chiang Mai University, Chiang Mai 50100, Thailand; 5 Center of Excellence in Microbial Diversity and Sustainable Utilization, Chiang Mai University, Chiang Mai 50100, Thailand; 6 Office of Research Administration, Chiang Mai University, Chiang Mai 50200, Thailand

**Keywords:** *Schizophyllum commune* exopolysaccharides, *Salmonella*, non-typhoidal salmonellosis, gut immunity, gut epithelium invasion, macrophages, immunomodulator, mushroom extract

## Abstract

Acute non-typhoidal salmonellosis (NTS) from non-typhoidal *Salmonella* remains a major cause of foodborne bacterial gastroenteritis, and non-antibiotic interventions are needed to combat multidrug-resistant NTS. Bioactive compounds from edible mushroom extracts have shown both direct and indirect antimicrobial activities on *Salmonella*. However, the variation in their antimicrobial activity could be due to several factors, including the extract’s form and strain. This study investigated the ability of crude exopolysaccharides (EPs) produced by *Schizophyllum commune* CMU-01 to limit *Salmonella* infection in vitro. Agar well diffusion and liquid culture were used to determine the direct anti-*Salmonella* activity of *S. commune* EPs, while the gentamicin protection assay and qPCR in human gut epithelium (T84 cells) and murine macrophages (RAW264.7 cells) were used to investigate its indirect (immunomodulatory) activity. Our data reveal that *S. commune* EPs do not confer the direct antimicrobial property against *Salmonella*. However, its immunomodulatory activity in two important components of the gut innate defense (the gut epithelium and macrophages) against *Salmonella* infection has been demonstrated. *S. commune* EPs reduce *Salmonella* gut epithelial cell invasion and activate macrophages toward M1 (inflammatory phenotype) polarization, resulting in the reduction in intracellular *Salmonella* burdens. Alterations in proinflammatory and anti-inflammatory cytokine gene expressions were also detected in *S. commune* EPs-treated cells. These findings suggest that the host innate immune response to fungal exopolysaccharides derived from *S. commune* CMU-01 favors reducing *Salmonella* proliferation within host cells by altering the expression levels of proinflammatory cytokines.

## 1. Introduction

Acute non-typhoidal salmonellosis (NTS), caused by ingestion of non-typhoidal *Salmonella*, remains one of the most common foodborne bacterial infections worldwide [1,2]. *Salmonella* is a genus of facultative anaerobic bacterium that belongs to the phylum Pseudomonadota and family Enterobacteriaceae. Consuming *Salmonella*-contaminated food or water is the primary route of non-typhoidal *Salmonella* transmission, followed by close contact with infected food-animal reservoirs, such as pigs or cattle [3]. Non-typhoidal *Salmonella* comprises more than 2500 serovars of *Salmonella enterica* subspecies I *enterica*. However, the most common serovars responsible for animal and human infections include Typhimurium, Enteritidis, Rissen, and Derby [4]. Even though most acute NTS patients with the proper supportive treatments could have self-limiting conditions, some individuals might develop a life-threatening condition termed invasive salmonellosis, especially those who are immunocompromised [5]. Misuse and overuse of multiple antibiotics in medical practice and agricultural activities over the past decades have led to the emergence of multidrug-resistant (MDR) non-typhoidal *Salmonella*, including a globally widespread carbapenem-resistant phenotype [6]. Hence, finding alternative interventions to antibiotics is an urgently needed strategy to tackle the rise in MDR *Salmonella*.

The antibacterial and immunomodulatory effects of edible mushroom extracts and metabolites have been mechanistically demonstrated in several studies. For example, phenolic compounds in methanol extract from the fruiting body of the basidiomycete *Coriolus versicolor*, (Turkey tail mushroom) can directly inhibit the growth of two human pathogenic bacteria, *Staphylococcus aureus* and *Salmonella enterica* serovar Enteritidis [7]. Exposure to *C. versicolor* methanol extract damages the *S.* Enteritidis cell envelope, particularly at the polar and septal regions of bacterial cells, due to the high concentration of the cardiolipin-rich domain [8]. The methanolic extract from the fruiting body of another edible basidiomycete, *Pleurotus ostreatus* (oyster mushroom), was found to have antimicrobial activity against several human pathogens (*S. aureus*, *Salmonella enterica* Typhi, *Acinetobacter* sp., and *Proteus mirabilis*) [9]. A quaternary ammonium compound conjugated with sesquiterpene, a bioactive volatile compound, in fermented mycelium of *Stereum hirsutum* (false turkey tail) mushroom showed the direct antibacterial activity against *Escherichia coli*, *S. aureus*, and *S.* Typhimurium [10].

While only a few studies have reported the direct antibacterial effect of mushroom extracts against *Salmonella*, the immunomodulatory properties (indirect effects) of edible mushrooms against bacterial infections, including *Salmonella*, have been more extensively investigated. For instance, the extracts rich in β-glucan from the fruiting body of edible medicinal mushroom *Hericium erinaceus* (Yambushitake mushroom) enhance macrophage killing activity against *S.* Typhimurium [11]. The *H. erinaceus* extract treatment increased macrophage inducible nitric oxide (NO) synthase (iNOS) mRNA expression and NO production. NO is an important substance for the bactericidal reactive nitrogen species (RNS) production in phagocytes. However, the anti-*Salmonella* effect of mushroom extracts may also vary among species and testing models.

*Schizophyllum commune* (split gill mushroom), a saprobic fungus belonging to the phylum Basidiomycota, is a medicinal and edible mushroom whose antibacterial and immunomodulatory activities have been reported in several previous studies [12,13,14,15]. Nonetheless, the study’s outcomes vary across different *S. commune* strains, parts, and forms. The direct antibacterial and anti-biofilm activities of the ethyl acetate extract of *S. commune* mycelial culture against *Salmonella* were demonstrated [13]. Exopolysaccharides (EPs) from a submerged mycelial fermentation of *S. commune* exhibit an anti-inflammatory effect by inhibition of iNOS production in macrophages [12]. The direct anti-*Salmonella* effect of the living *S. commune* BCC64 growing on the plate, collected from the Georgian mountain, was also illustrated [16]. In our previous study, we optimized EPs production from the culture broth of the submerged mycelial culture of *S. commune* CMU-01 and demonstrated its immunomodulatory activity in macrophages [17]. However, the direct anti-*Salmonella* effect and the contribution of *S. commune* CMU-01 EPs on *Salmonella* gut invasion, an important step in *Salmonella* pathogenesis, have never been investigated. Thus, we reported here both the direct and indirect roles of *S. commune* CMU-01 EPs against *Salmonella* infection using a monolayer of human colonic epithelium (T84) and murine macrophage (RAW264.7) cell lines.

## 2. Results

### 2.1. Carbohydrate Patterns of S. commune CMU-01 by FT-IR Analysis

Our previous study has already demonstrated that the crude fungal EPs of *S. commune* CMU-01 exhibited a typical carbohydrate pattern similar to that of schizophyllan [17]. Here, we re-analyzed the FT-IR spectra of crude *S. commune* CMU-01 EPs used in this study (Figure S1). The spectral analysis revealed several key functional groups, including broad peaks at 3253.8–3280.4 cm^−1^, indicating hydroxyl (–OH) stretching vibrations, a primary characteristic of polysaccharide chains. Peaks at 2885.9 to 2918.4 cm^−1^ corresponded to C–H stretching vibration of aldehyde groups and saturated carbon, while absorption peaks at 1627.8 to 1634.3 cm^−1^ were assigned to carbonyl (C=O) group and amide (RCONH_2_) C=N stretching vibration. A characteristic absorption at 1361.7 to 1367.1 cm^−1^ showed bending vibration of C–H and C–C stretching in aliphatic chains. Furthermore, the strong absorbance between 994.4 and 1028.9 cm^−1^ revealed unique absorption peaks characteristic of polysaccharides. These peaks specifically demonstrated the stretching vibrations of C–O, C–O–C, and C–O–H linkages, which are associated with glycosidic bonds. The detailed peak characteristics are summarized in Table 1.

### 2.2. Crude S. commune CMU-01 EPs Can Be Used as a Nutrient Source for Salmonella

**Figure 1:**
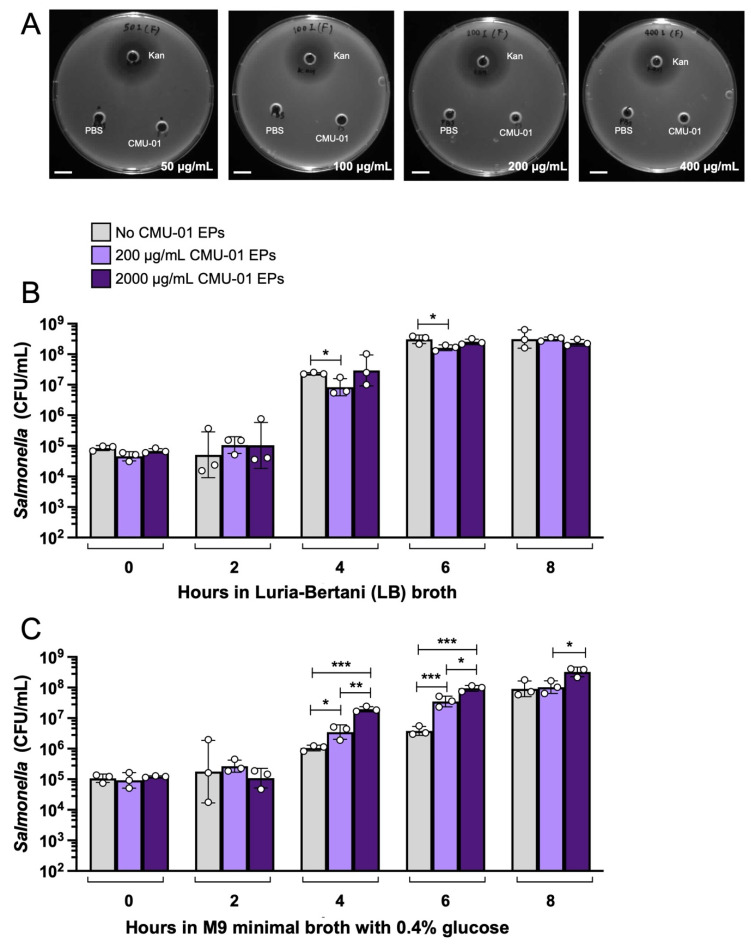
No direct anti-*Salmonella* effect of *S. commune* CMU-01 EPs. No zone of inhibition was observed in any concentrations of *S. commune* CMU-01 EPs (50, 100, 200, and 400 µg/mL) (**A**). Minimal inhibition of *Salmonella* growth was observed in LB broth supplemented with 200 µg/mL *S. commune* CMU-01 EPs at 4 and 6 h (**B**). *S. commune* CMU-01 EPs promotes dose-dependent growth of *Salmonella* in an M9 minimal medium with 0.4% glucose at 4, 6, and 8 h (**C**). Bars represent the geometric mean, with geometric standard deviation of three independent experiments. * *p* < 0.05; ** *p* < 0.01; *** *p* < 0.001. The indicated scale bar in (**A**) is 1.0 cm. Kan, 0.05 mg/mL kanamycin (AppliChem, Darmstadt, Germany); PBS, phosphate-buffered saline.

The direct anti-*Salmonella* activity of *S. commune* CMU-01 EPs was assessed using an agar well diffusion assay with kanamycin antibiotic as a positive control (Figure 1A). Surprisingly, no clear zone was observed around the well at any of the tested EP concentrations (50, 100, 200, and 400 µg/mL). Then, the kinetics of *Salmonella* growth in Luria–Bertani (LB) broth, with or without crude *S. commune* CMU-01 EPs, were determined. We found a minimal reduction in *Salmonella* CFU/mL in the 200 µg/mL *S. commune* EPs-treated group at 4 and 6 h, but not in the 2000 µg/mL-treated group (Figure 1B). To investigate the possible role of *S. commune* CMU-01 EPs as a nutrient source for *Salmonella*, we used M9 minimal broth supplemented with 0.4% glucose. Our data show that *S. commune* CMU-01 EPs enhances *Salmonella* growth at 4, 6, and 8 h in a dose-dependent manner (Figure 1C).

### 2.3. Gut Epithelial and Macrophage Cytotoxicity Test of S. commune CMU-01 EPs

**Figure 2:**
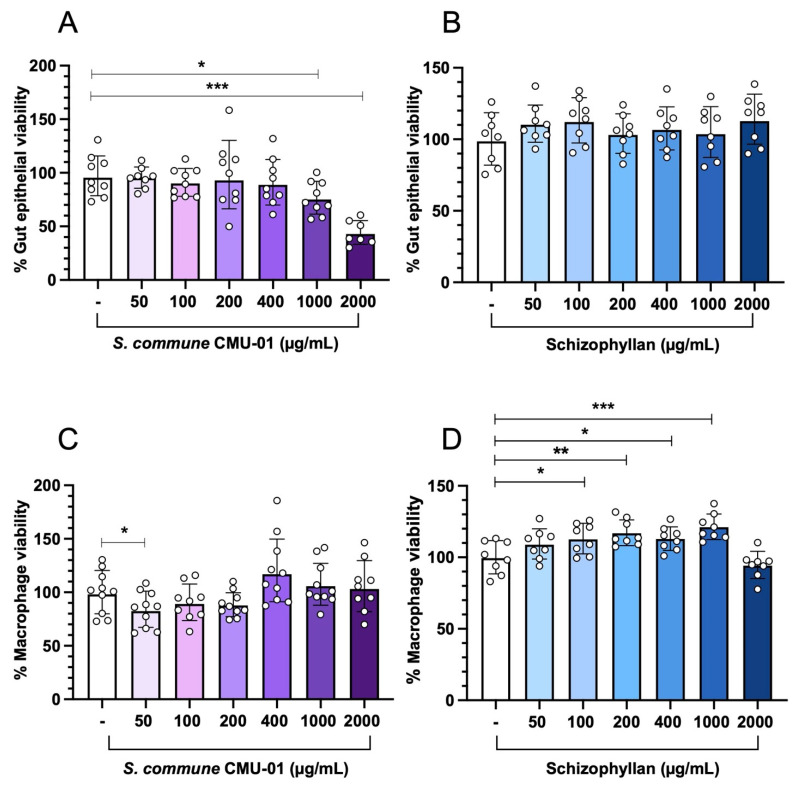
Percentage of host cell viability in different *S. commune* EPs concentration by an MTT assay. High doses (1000 and 2000 µg/mL) of *S. commune* CMU-01 EPs decreased human colonocyte (T84) cell viability (**A**) but not for murine macrophage (RAW264.7) (**C**). Commercially available schizophyllan (Biosynth^®^) EPs did not decrease the viability of both T84 and RAW264.7 cells (**B**,**D**). Bars represent the geometric mean with geometric standard deviation of at least eight independent experiments. * *p* < 0.05, ** *p* < 0.01, *** *p* < 0.001.

Next, we compared the cytotoxicity of different concentrations of crude *S. commune* CMU-01 EPs and the commercially available schizophyllan on the cell lines of human colonic epithelium (T84) and murine macrophage (RAW264.7) by MTT assay (Figure 2). The viability of T84 cells decreased when pretreated with 1000 and 2000 µg/mL of crude *S. commune* CMU-01 EPs, but not with schizophyllan (Figure 2A and Figure 2B, respectively). For RAW264.7 cells, the only minimal reduction in cell viability was observed in the 50 µg/mL crude *S. commune* CMU-01 EPs-pretreated cells (Figure 2C). Interestingly, pretreatment with schizophyllan increased cell viability in RAW264.7 cells (Figure 2D).

### 2.4. S. commune CMU-01 EPs Reduce Salmonella Invasion to Human Gut Epithelium and Increase Mouse Macrophage Function

**Figure 3:**
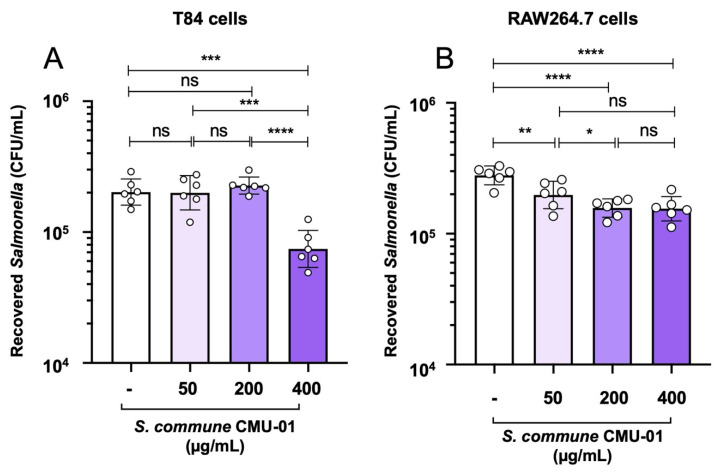
Dose-dependent effect of *S. commune* CMU-01 EPs in the reduction in *Salmonella* invasion to human colonocyte (T84) and activation of phagocytic activity of murine macrophage (RAW264.7). T84 and RAW264.7 cells were pretreated with three concentrations of *S. commune* EPS for 24 h prior to being infected with *S.* Typhimurium (MOI = 1) (**A**,**B**). Then, the gentamicin protection assay was used to determine the intracellular *Salmonella* counts (CFU/mL). Bars represent the geometric mean, with geometric standard deviation. * *p* < 0.05; ** *p* < 0.01; *** *p* < 0.001; **** *p* < 0.0001; ns, a non-statistically significant difference.

A gentamicin protection invasion assay for 3 h of *Salmonella* infection was used to determine the number of *S.* Typhimurium that invaded and survived within both gut epithelial (T84) and murine macrophage RAW264.7 cells (Figure 3). Gut epithelial cells, especially the colonic enterocytes, are an important host cell type encountered by *Salmonella* in the gut lumen. Intestinal barrier integrity and innate immune signaling originating from the gut epithelium are necessary for gut immunity against *Salmonella* infection in vivo. Macrophages are specialized white blood cells that can engulf and kill invading *Salmonella* through phagocytosis. Here, we investigate the role of pretreating both cells with *S. commune* CMU-01 EPs in reducing the intracellular population of *S.* Typhimurium. Comparison of the recovered *Salmonella* numbers (CFU/mL) with the untreated with *S. commune* CMU-01 EPs as a control, our data show that high doses (400 µg/mL) of *S. commune* CMU-01 EPs significantly reduce *S.* Typhimurium invasion into T84 cells (Figure 3A). All three doses (50, 200, and 400 µg/mL) of *S. commune* CMU-01 EPs significantly reduce intracellular *S.* Typhimurium burdens in macrophages (Figure 3B).

### 2.5. Immunomodulatory Effect of S. commune CMU-01 EPs on Human Gut Epithelium

**Figure 4:**
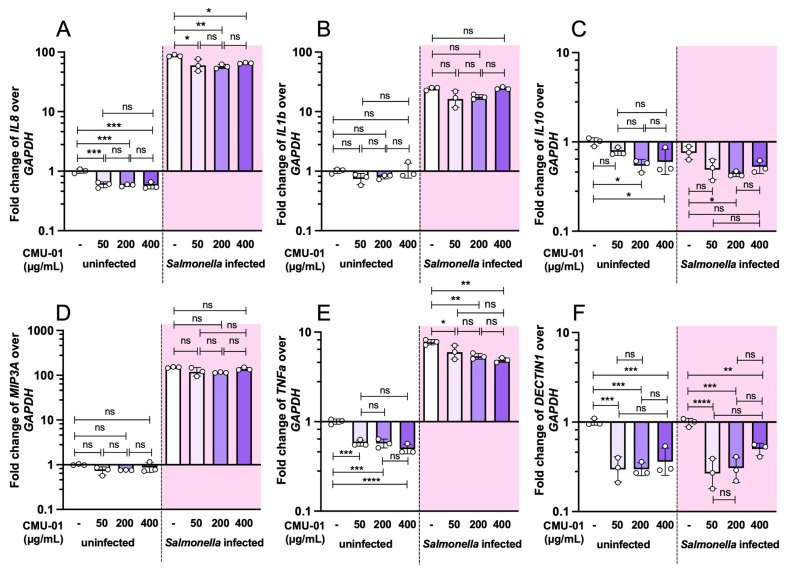
Immunomodulatory effect of *S. commune* CMU-01 EPs on human gut epithelium with and without *S.* Typhimurium infection. Human gut epithelium (T84) cells were pretreated with different concentrations of *S. commune* CMU-01 EPs for 24 h before *Salmonella* infection (MOI = 1) (shaded area) or phosphate-buffered saline (PBS) (non-shaded area). Fold changes in cytokine mRNA expressions (*IL8*, *IL1b*, *IL10*, *MIP3A*, *TNFa*, and *DECTIN1*) were determined by quantitative PCR (**A**–**F**). Bars represent the geometric mean with geometric standard deviation of three independent experiments. * *p* < 0.05, ** *p* < 0.01, *** *p* < 0.001, **** *p* < 0.0001; ns, a non-statistically significant difference.

Three different doses (50, 200, and 400 µg/mL) of *S. commune* CMU-01 EPs were used to pretreat T84 cells for 24 h before *S.* Typhimurium infection. The fold change in immune-related gene expressions was measured by qPCR. *S. commune* CMU-01 EPs did not induce the mRNA expressions of major proinflammatory cytokines: interleukin (IL)-8, IL-1β, macrophage inflammatory protein-3 alpha (MIP-3A), and tumor necrosis factor-alpha (TNF-α) in the absence of *S.* Typhimurium infection (Figure 4). Interestingly, pretreatment with *S. commune* CMU-01 EPs significantly reduced *IL8* and *TNFα* expressions in T84 cells (Figure 4A and Figure 4E, respectively). No change in *IL1β* and *MIP3A* expressions was detected in *S.* Typhimurium-infected T84 cells (Figure 4B and Figure 4D, respectively). However, pretreatment with *S. commune* CMU-01 EPs markedly reduces mRNA transcript expression of anti-inflammatory cytokine *IL10* and *DECTIN1*, the pattern-recognition receptor (PRR) to fungal β-glucans, in T84 cells regardless of *S.* Typhimurium infection (Figure 4C and Figure 4F, respectively).

### 2.6. S. commune CMU-01 EPs Enhances Proinflammatory Cytokine mRNA Expression in Macrophages

**Figure 5:**
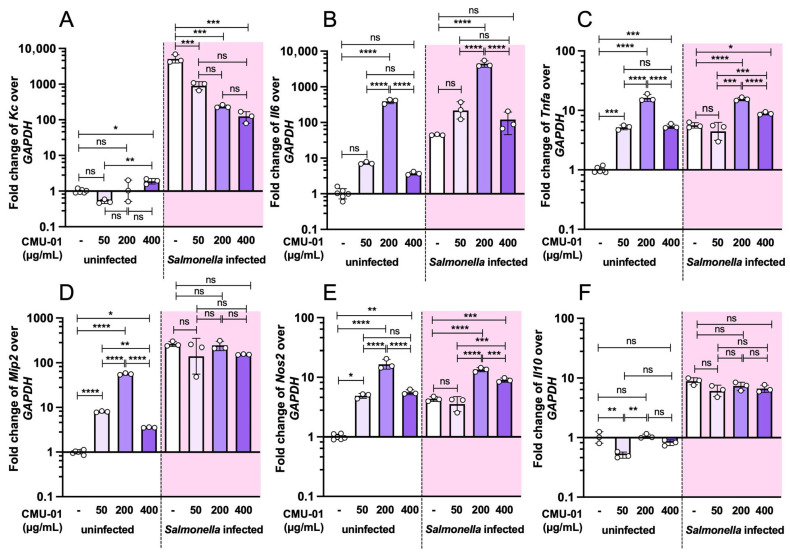
Immunomodulatory effect of *S. commune* CMU-01 EPs on murine macrophage with and without *S.* Typhimurium infection. Murine macrophage (RAW264.7) cells were pretreated with different concentrations of *S. commune* CMU-01 EPs for 24 h before *Salmonella* infection (MOI = 1) (shaded area) or PBS (non-shaded area). Fold changes in cytokine mRNA expressions (*Kc*, *Il6*, *Tnfa*, *Mip2*, *Nos2*, and *Il10*) were determined by quantitative PCR (**A**–**F**). Bars represent the geometric mean with geometric standard deviation of three independent experiments. * *p* < 0.05, ** *p* < 0.01, *** *p* < 0.001, **** *p* < 0.0001; ns, a non-statistically significant difference.

Our previous study demonstrated that low doses of *S. commune* CMU-01 EPs (50 and 200 µg/mL) enhance phagocytic activity of mouse macrophage (RAW264.7) against *S.* Typhimurium infection [17]. Here, we further investigate the dose-dependent effects of *S. commune* CMU-01 EPs on RAW264.7 cells in response to *S.* Typhimurium infection (Figure 5). Three different doses (50, 200, and 400 µg/mL) of *S. commune* CMU-01 EPs were used to pretreat mouse macrophages for 24 h before *S.* Typhimurium infection. Our data reveal that pretreatment with *S. commune* CMU-01 EPs upregulates the expression of the proinflammatory cytokine genes *Il6*, *Tnfα*, *Mip2 (or Cxcl-2)*, and *Nos2* in RAW264.7 cells. However, pretreatment of *S. commune* CMU-01 EPs significantly reduced chemotactic cytokine keratinocyte-derived cytokine (*Kc*) or mouse chemokine (C-X-C-motif) ligand-1 *(Cxcl-1)* expression in *S.* Typhimurium-infected RAW64.7 cells (Figure 5A).

### 2.7. S. commune CMU-01 EPs Activate Macrophages Towards M1-Polarization

**Figure 6:**
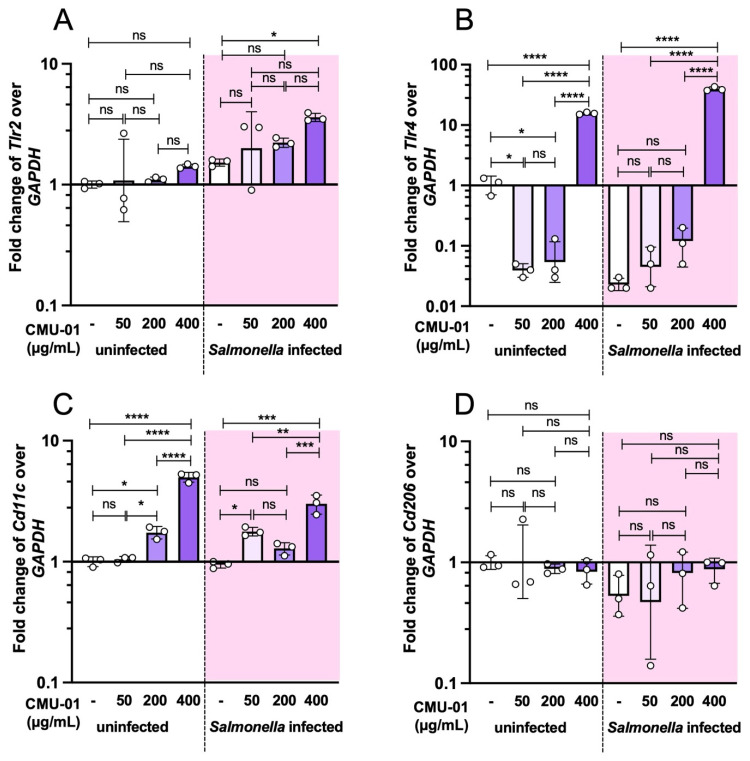
Effect of *S. commune* CMU-01 EPs on the expressions of cell surface marker genes of macrophage with and without *S.* Typhimurium infection. Murine macrophage cells were pretreated with *S. commune* CMU-01 EPs for 24 h before *Salmonella* infection (MOI = 1) (shaded area) or PBS (non-shaded area). The mRNA expression fold changes in cell surface receptors and markers (*Tlr2*, *Tlr4*, *Cd11c*, and *Cd206*) were determined by quantitative PCR (**A**–**D**). Bars represent the geometric mean with geometric standard deviation of three independent experiments. * *p* < 0.05, ** *p* < 0.01, *** *p* < 0.001, **** *p* < 0.0001; ns, a non-statistically significant difference.

Since PRRs play a crucial role in the innate immune response to antigens across several immune cell types, including macrophages, we measured the expression of the important innate PRRs, Toll-like receptors (TLRs) 2 and 4, in macrophages pretreated with *S. commune* EPs (Figure 6A and Figure 6B, respectively). Pretreatment of RAW264.7 cells with low doses (50 and 200 µg/mL) of *S. commune* CMU-01 EPs significantly downregulated *Tlr4*. Nonetheless, pretreatment with high dose (400 µg/mL) of *S. commune* CMU-01 EPs markedly upregulated *Tlr4* with or without *S.* Typhimurium infection. Interestingly, *S. commune* CMU-01 EPs did not significantly affect *Tlr2* expression in macrophages. The polarization of macrophage between M1 and M2 subpopulations was detected by the mRNA expression of macrophage M1 and M2 marker genes *Cd11c* and *Cd206*, respectively, as previously performed [18]. Our data reveal that pretreatment with *S. commune* CMU-01 EPs for 24 h significantly upregulated *Cd11c* expression but not *Cd206*, indicating that *S. commune* CMU-01 EPs induce a phenotypic shift in macrophages toward the M1 subpopulation.

## 3. Discussion

Antibiotic resistance has become a major problem in several previously treatable bacterial infectious diseases, including non-typhoidal salmonellosis (NTS). The emergence of the MDR phenotype in non-typhoidal *Salmonella* strains has been reported worldwide [3,4,19,20]. Thus, alternatives to antibiotics for NTS are urgently needed. In addition to directly killing the pathogen, stimulating the host immune response is essential for combating *Salmonella* infection in vivo. For centuries, edible and medicinal mushrooms have been widely known for their antimicrobial and immunomodulatory activities, with minimal adverse effects on the human host [21,22,23]. Several groups of bioactive compounds in mushrooms exhibit antimicrobial and immunomodulatory activities, including terpenes, terpenoids, lectin, fungal immunomodulatory proteins (FIPs), and polysaccharides. However, the distributions of these compounds vary among mushroom species, forms (extract or whole mushroom), extract formulations, and strains, which play a significant role in their beneficial effects [24,25]. Among the several types of medicinal mushrooms, *Schizophyllum commune* (*S. commune*) is one of the most widely studied for its antimicrobial and immune-enhancing properties [26]. However, the contribution of *S. commune* exopolysaccharides (EPs) in enhancing gut immunity against *Salmonella* infection remains poorly studied. In this study, we reported both the direct and indirect anti-*Salmonella* mechanisms of the water-soluble *S. commune* crude EPs in vitro (reducing *Salmonella* invasion of the gut epithelium and enhancing macrophage function).

The crude EPs were extracted from the culture broth of the submerged mycelial culture of *S. commune* CMU-01. The FT-IR analysis confirmed the typical carbohydrate pattern, with the presence of both alpha and beta glycosidic bonds in *S. commune* CMU-01 EPs, similar to schizophyllan, as previously reported [17]. Schizophyllan is an exopolysaccharide β-(1,3)(1,6)-glucan produced by *S. commune* and has been reported to have immunomodulatory properties against some bacterial and viral infections [27,28]. This suggests that crude *S. commune* CMU-01 EPs should confer the beneficial effects of schizophyllan against the bacterial infection. Although the FT-IR spectra of *S. commune* CMU-01 EPs showed carbohydrate functional group patterns similar to those reported for commercial schizophyllan, the FT-IR analysis alone is still insufficient to confirm structural equivalence. Moreover, mushroom crude EPs may contain additional bioactive compounds or structural variations that could influence their biological activities. Further studies are required to elucidate the detailed structures of *S. commune* CMU-01 EPs using advanced analytical techniques, such as methylation analysis, nuclear magnetic resonance (NMR), and mass spectrometry, to better understand their structural characteristics and potential biological activities.

The direct anti-*Salmonella* activity of *S. commune* CMU-01 EPs was determined using agar diffusion and liquid-media growth assays. Surprisingly, our data reveal that *S. commune* CMU-01 EPs do not exhibit the direct antimicrobial activity against *S.* Typhimurium in either solid or liquid media. In contrast, *S. commune* CMU-01 EPs could serve as a nutrient source for *S.* Typhimurium, especially under nutrient-limited conditions. In the liquid-media growth assay, our data exhibit the direct minimal reduction in *Salmonella* growth in LB broth observed only in low dose (200 µg/mL of *S. commune* CMU-01 EPs) at 4 and 6 h, which might be due to the variation in the relatively small size of the experimental group (n = 3). However, this mild inhibitory effect of low-dose *S. commune* CMU-01 EPs was not observed in the nutrient-poor medium (M9 minimal broth with 0.4% glucose). In the deprivation of enriched nutrients like LB broth, *Salmonella* use *S. commune* CMU-01 crude EPs as an extra nutrient source to support their growth during the exponential phase (4–8 h). The dose-dependent effect of *S. commune* CMU-01 crude EPs as a nutrient source for *Salmonella* was also shown. In contrast to our findings, Sharma A. et al. recently reported that ethyl acetate extracts of *S. commune* Sch1 confer strong antimicrobial activity against several human pathogens, including *Salmonella enterica* [13]. This difference might be due to a different protocol being used. In the study by Sharma et al., the plates in the agar diffusion assay were initially pre-chilled at 4 °C for 4 h to allow the inhibitors in Sch1 extract to diffuse into the nutrient agar.

Mild toxicity of *S. commune* CMU-01 EPs toward human gut epithelial cell (T84) viability was observed in our study. Only high doses (1000 and 2000 µg/mL) of *S. commune* CMU-01 reduce human gut epithelial cell viability, but not schizophyllan. This reduction in cell viability caused by crude *S. commune* CMU-01 EPs may be due to the presence of other unidentified compounds. *S. commune* CMU-01 EPs and schizophyllan do not reduce the viability of mouse macrophages (RAW264.7 cells). These findings are consistent with our previous report [17]. However, in this study, we found a mild reduction in the percentage of RAW264.7 viability at 50 µg/mL crude *S. commune* CMU-01 treatment, but not in T84 cells. Interestingly, this result is inconsistent with our previous report, which showed no reduction in the viability of RAW264.7 cells treated with crude *S. commune* CMU-01 for 24 h [17]. This suggests the possibility of large variation in an MTT assay for crude fungal EPs. More sample sizes should be considered in future MTT assay experiments.

Here, we found that pretreatment with schizophyllan (100–1000 µg/mL) enhances the viability of a macrophage cell line. The positive effect of fungal polysaccharides on macrophage (RAW264.7) viability and proliferation has been previously demonstrated. For example, the water-soluble polysaccharide of medicinal fungus *Morchella sextelata* significantly promotes cell proliferation, phagocytosis, and nitric oxide production of RAW264.7 cells [29]. EPs from *Cordyceps cicadae* also exhibit RAW264.7 cell proliferation and phagocytosis. In high-dose polysaccharide-treated cells, cell proliferation might be reduced due to increased osmotic pressure from added polysaccharides in the cell culture medium [30]. However, the mechanism underlying the activation of cell proliferation by fungal polysaccharides remains unclear.

Our previous study has demonstrated that *S. commune* CMU-01 EPs can significantly enhance macrophage function against *Salmonella* infection in the cell line model. Here, we reported the dose-dependent manner of *S. commune* CMU-01 EPs on two important gut immune cells, using gut epithelial and macrophage cell lines. A dose-dependent effect of *S. commune* CMU-01 EPs in reducing *Salmonella* invasion of gut epithelial cells and enhancing macrophage function is reported. However, due to the limitation of the cell invasion experiments in our study, the role of *S. commune* CMU-01 EPs in other steps of phagocytosis (e.g., attachment and engulfment) still cannot be ruled out. For example, the reduction in *Salmonella* numbers observed in our study (Figure 3) could result from *S. commune* CMU-01 EPs directly blocking *Salmonella* attachment to T84 and RAW264.7 cells. To better understand the mechanism by which *S. commune* CMU-01 EPs reduce gut epithelial invasion and enhance macrophage function against *Salmonella* infection, further experiments are warranted. For instance, centrifugation of a cell culture monolayer (600× *g* for 5 min) after adding *Salmonella* should be used in the gut epithelial attachment study. This step could increase the likelihood of *Salmonella* attachment to the gut epithelium [31].

The mechanism by which fungal polysaccharides directly reduce *Salmonella* invasion of the gut remains poorly understood. In chickens, the *S. commune* β-glucan reduces *Salmonella* colonization of gut tissues and systemic organs by enhancing the phagocytic activity of abdominal macrophages, without directly blocking gut invasion [32]. For human gut epithelium immune modulation, *S. commune* CMU-01 EPs reduce proinflammatory cytokine (IL-8 and TNF-α) mRNA expressions with a mild reduction in the anti-inflammatory cytokine IL-10. This suggests different immunomodulatory activities of *S. commune* CMU-01 EPs on various cytokines produced by the human gut epithelium.

The molecular interaction between the host innate immune receptor (pattern recognition receptor, PRR) and microbial-specific molecules (microbial-associated molecular patterns, MAMPs) is the key step of the innate immune response. Dectin-1 is an innate PRR for fungal β-glucans and possesses immunostimulatory and antitumor activities in other models, as reviewed in Batbayar S. et al. [33]. Exposure of *S. commune* β-glucan to innate immune cells induces a proinflammatory pathway through the dectin-1 and nuclear factor (NF)-kβ-dependent manner [12]. The dectin-1 receptor system has been reported as a significant pathway in higher animals for protection against fungal invasion [33]. The immunostimulatory signaling from the binding of fungal β-glucan to host innate immune cells, including macrophages and dendritic cells, could occur via the dectin-1 signal transduction pathway. In this study, we found a reduction in *DECTIN1* mRNA levels in the human colonic cell line (T84), regardless of *Salmonella* infection. This may be due to lower *DECTIN1* expression in the large intestinal epithelium than in the small intestine or in other innate immune cells, such as macrophages or dendritic cells.

*S. commune* CMU-01 EPs significantly enhance the macrophage function against *Salmonella* infection by increasing the expression of the *Nos2*, *IL6*, and *Tnfα*. These enzymes and cytokines play a role in the production of antimicrobial substances such as RNS and reactive oxygen species (ROS) of macrophages. These results are consistent with a previous report [12]. However, this study detected only changes in mRNA levels, not in protein levels, which might be affected by post-transcriptional modifications.

Fungal β-glucans can act as activators of both M1 (classically activated) and M2 (alternatively activated) macrophages, depending on the context and microenvironment, as recently reviewed [34]. However, most reports show that mushroom extract preferentially promotes M-1 polarization. For instance, a mycelium-derived aqueous extract of *Pleurotus ostreatus* mushroom increases proinflammatory cytokine TNF-α and IL-6 production, and also enhances phagocytic function in macrophages [9]. *S. commune* CMU-01 EPs preferentially activate macrophages toward M1 polarization by the upregulation of *Cd11c* and *Tlr4*, while no alteration in M2 (alternatively activated macrophages) *Cd206* mRNA.

The role of mushroom extract in the host innate immune response has been investigated in several animal models, which is the major limitation of this in vitro study. For example, Kim S. et al. demonstrated that the fermented rice bran extract from *Lentinus edodes* (shiitake mushroom) liquid mycelial culture exhibits anti-*Salmonella* mechanisms by increasing phagocytosis of extracellular bacteria, autophagic capture of intracellular bacteria, and preventing *Salmonella* translocation across the gut epithelium to the mouse spleen and liver [35]. The aqueous extract from *Agaricus brasiliensis* (royal sun mushroom) did not alter the level of SIgA, serum TNF-α, Interferon gamma (IFN-γ), and IL-10 level in *Salmonella*-infected mice treated with the extract [36]. On the contrary, the aqueous extract of *A. brasiliensis*, rich in carbohydrates (β-glucans), proteins, and phenolic compounds, increased the survival of septic mice in the cecal ligation and puncture murine sepsis model. *A. brasiliensis* exhibited immunomodulatory and antioxidant properties, by increasing the antioxidant status of septic mice and reducing oxidative stress markers in septic mice [37]. Thus, the immunomodulatory property of *S. commune* CMU-01 against *Salmonella* should be further investigated in a proper animal model, such as a streptomycin-pretreated mouse colitis model of acute NTS.

## 4. Materials and Methods

### 4.1. Ethical Approvals

The study was approved by the Institutional Biosafety Committee of the Faculty of Medicine, Chiang Mai University (Approval No. 02014/2564). No animal or human studies are involved in this study.

### 4.2. Fungal Mycelial and Crude Exopolysaccharide Preparation

Schizophyllum commune CMU-01 was obtained from the culture collection of the Research Center of Microbial Diversity and Sustainable Utilization, Faculty of Science, Chiang Mai University, Thailand. The fungal mycelia were cultivated on potato dextrose agar (PDA; Conda, Madrid, Spain) and incubated at 30 °C for 7 days. Then, the mycelial plugs (about 5 mm in diameter) were inoculated into 80 mL of potato dextrose broth (PDB, pH 6.0) on the reciprocal shaker (NR-10 Bioshaker^®^, Koshigaya, Japan) at 110 rpm for 14 days at 28 ± 2 °C. The fungal mycelia were filtered using a 25 µm membrane filter (Miracloth, Merck Millipore, Burlington, MA, USA). Subsequently, the crude EPs in the culture filtrate were precipitated by adding an equal volume of 75% (*v*/*v*) ethanol at 4 °C for 24 h. The mixture was then centrifuged at 10,000× *g* for 15 min. After washing three times with 95% ethanol, the EPs were dissolved in deionized water, freeze-dried in a lyophilizer at −50 °C (FreeZone 2.5 Liter Benchtop Freeze Dryer, Labconco, Kansas City, MO, USA) until completely dry, and stored at −20 °C until further use (Supplementary Table S1 and Figure S1A).

### 4.3. Fourier Transform-Infrared Spectroscopy (FT-IR) Analysis

Structural and functional groups of *S. commune* CMU-01 EPs were determined by the Fourier transform-infrared (FT-IR) spectroscopy at the Science and Technology Service Center, Faculty of Science, Chiang Mai University, Thailand. The FT-IR analysis was performed as previously described, with slight modifications [17]. In brief, the sample was prepared by mixing fungal EPs with spectroscopic-grade potassium bromide (KBr) at a 1:100 ratio in a dry mortar, grinding to a fine powder, and pressing into a disk for analysis. The absorption was measured and analyzed using a FT-IR spectrophotometer (Thermo Fisher Scientific, Waltham, MA, USA) in the wavenumber range between 400 and 4000 cm^−1^. The schizophyllan (Biosynth, Newbury, UK) was used as a standard.

### 4.4. Bacterial Strain and Cultivation

The bacterial strain used in this study is shown in Supplementary Table S1. The nalidixic acid-resistant derivative of *Salmonella enterica* serovar Typhimurium IR715 (ATCC 14028) was grown in Luria–Bertani (LB) broth (10 g/L tryptone, 5 g/L yeast extract, and 10 g/L NaCl) (Difco, Sparks, MD, USA) with shaking at 37 °C for 16–18 h under the ambient atmospheric gas conditions. The 0.05 mg/mL nalidixic acid (AppliChem, Darmstadt, Germany) was added to the culture medium.

### 4.5. Agar Well Diffusion Assay

Freeze-dried *S. commune* CMU-01 EPs were dissolved in sterile 1× phosphate-buffered saline (PBS) to obtain the concentrations of 50, 100, 200, and 400 μg/mL. The mixtures were filtered through a 0.45 µm pore-size filter (Puradisc, Cytiva, Amersham, UK). The agar well diffusion assay was performed as previously described with a slight modification [38]. In short, the overnight culture of *S. enterica* Typhimurium strain IR715 was added to LB broth (Difco, Sparks, MD, USA) with 0.75% agar (Criterion, Hercules, CA, USA) at a 1:100 ratio. Then, 20 mL of the mixture was poured into a 90 × 15 mm sterile Petri dish. Once the agar had solidified, 6 mm wells were created and filled with 60 µL of the *S. commune* CMU-01 EPs solution. 0.05 mg/mL kanamycin (AppliChem, Darmstadt, Germany) and 1X PBS were used as positive and negative controls, respectively. The significant anti-*Salmonella* effect was interpreted by the presence of an inhibition (clear) zone around each well with a diameter larger than 1 mm after incubation at 37 °C for 16 h.

### 4.6. Bacterial Growth Assay in Enriched and Minimal Liquid Media

The overnight culture of *Salmonella* in LB broth was diluted 100 times. Then, 100 µL of the diluted culture was inoculated into 10 mL of LB (enriched) broth or M9 minimal medium (for 100 mL: 20 mL 5X M9 salts (Na_2_HPO_4_·7H_2_O, KH_2_PO_4_, NaCl, and NH_4_Cl), 1 M MgSO_4_, and 1 M CaCl_2_) with 0.4% glucose. Two concentrations of *S. commune* CMU-01 EPs (200 and 2000 µg/mL) were added to the bacterial culture, with PBS as the control. At 0, 2, 4, 6, and 8 h after inoculation, 300 µL of the bacterial culture was collected to enumerate *Salmonella* colony-forming units (CFU)/mL using a standard serial dilution technique and plating on agar plates with nalidixic acid.

### 4.7. Cell Viability Test by MTT Assay

Cell viability of T84 and RAW264.7 cells exposed to EPs was assessed using the MTT assay as previously described [38]. Briefly, the cells were seeded into a 96 well-plate with the density of 4 × 10^4^ cells/well. After 24 h, the cells were treated with different concentrations of EPs (*S. commune* CMU-01) and schizophyllan (Biosynth^®^, Newbury, UK) at 50, 100, 200, 400, 1000, and 2000 μg/mL for 24 h. Then, the culture media were discarded, and 300 μL of 1 mg/mL 3-(4,5-dimethyl-thiazol-2-yl)-2,5-diphenyltetrazolium bromide (MTT; AppliChem, Darmstadt, Germany) in PBS was added to each well. After 3 h of incubation at 37 °C with 5% CO_2_, MTT solution was discarded. To dissolve the purple-blue formazan precipitate generated by MTT, 150 μL of dimethyl sulfoxide (DMSO) (Sigma-Aldrich, Saint Louis, MO, USA) was added. The plate was then orbitally shaken (Nedtex, Taipei, Taiwan) for 10 min in the dark at room temperature. The absorbance was determined at 492 nm by a microplate reader (BioTek Synergy H4 Hybrid Reader, BioTek Instruments Inc., San Diego, CA, USA). The percentage of cell viability was calculated using the formula: cell viability (%) = [(Abs 492 nm of the treated group − blank)/(Abs 492 nm of the control − blank)] × 100.

### 4.8. Cell Culture Conditions

Human colonic epithelium T84 (ATCC; CCL-248) and murine macrophage RAW264.7 (ATCC; TIB-71) cells were purchased from the American Type Culture Collection (ATCC, Manassas, VA, USA). The completed growth medium for T84 cells contains Dulbecco’s Modified Eagle Medium (DMEM)/F12 (Cytiva, Logan, UT, USA) containing 2.5 mM L-Glutamine and 15 mM HEPES buffer, 5% heat-inactivated fetal bovine serum (FBS) (Cytiva, Logan, UT, USA), and 100 U/mL penicillin, and 0.1 g/mL streptomycin (Pen-Strep) (Cytiva, Logan, UT, USA). The completed growth medium for murine macrophage (RAW264.7, ATCC; TIB-71) comprised DMEM with 4 mM L-glutamine, 4500 mg/L glucose, and sodium pyruvate (Cytiva, Logan, UT, USA) supplemented with 10% heat-inactivated FBS, 100 U/mL penicillin, and 0.1 g/mL streptomycin (Pen-Strep) (Cytiva, Logan, UT, USA). Both cells were grown in a T75 flask at 37 °C with 5% CO_2_ under high-humidity conditions until the cell density reached the optimal level for the assay.

### 4.9. Gentamicin Protection Assay for Gut Epithelial Invasion and Macrophage Function

The assays were performed as previously reported, with a slight modification [39]. In short, T84 and RAW264.7 cells were used in gut epithelial invasion and phagocytic function, respectively. Cells were seeded into a 24-well plate at a density of approximately 10^5^ cells/well and cultured for 24 h. Then, the media was replaced with new complete media without FBS or antibiotics. After 24 h of incubation at 37 °C with 5% CO_2_, the cells were pretreated with EPs for 24 h and subsequently infected with 10^6^ CFU/well *Salmonella enterica* Typhimurium IR715 (multiplicity of infection (MOI) 10 for 3 h. The cells were washed twice with Dulbecco’s phosphate-buffered saline (DPBS) (Cytiva, Singapore) and treated with 100 μg/mL gentamicin sulfate (AppliChem, Darmstadt, Germany) in the cell culture medium for 90 min, then incubated at 37 °C with 5% CO_2_ to eliminate extracellular bacteria. The infected cells were washed twice with DPBS and lysed with 1% Triton-X-100 in PBS (AppliChem, Darmstadt, Germany) to collect the cell lysate for subsequent bacterial burden enumeration by using a standard plating technique. Recovered numbers of the intracellular population of *Salmonella* from each group were reported as CFU/mL.

### 4.10. Detection of Gene Expressions by Quantitative Polymerase Chain Reaction (qPCR)

T84 and RAW264.7 cells were cultivated in a 6-well plate at a density of approximately 10^6^ cells/well for 24 h. Then, cells were synchronized by growing them in the media without antibiotics and FBS for 24 h. The cells were pretreated with EPs and incubated at 37 °C with 5% CO_2_ for 24 h. Each well of EPs-pretreated cells was infected with 10^7^ CFU *S.* Typhimurium (MOI 10) for 3 h. Then, the cell culture media were discarded, and 1 mL TRIzol reagent (Invitrogen, Carlsbad, CA, USA) was added to each well to collect the cell lysate for subsequent RNA extraction. Total RNA from the cell lysate was extracted using a TRIzol reagent following the manufacturer’s guidelines. The mRNA was then converted to cDNA by using RevertAid First Strand cDNA reagents (Thermo Fisher Scientific, Vilnius, Lithuania). The qualitative polymerase chain reaction (qPCR) was done using the SensiFAST SYBR Lo-ROX Kit (Meridian Bioscience, Memphis, TN, USA) in ViiA 7 Real-Time PCR system (Applied Biosystems, Carlsbad, CA, USA). The list of primers used in this study is illustrated in Supplementary Table S2. The comparative Ct method (2^−ΔΔCt^) was used to evaluate the fold change in the target genes, with human *GAPDH* and mouse *Gapdh* as the housekeeping genes, as previously described [40].

### 4.11. Statistical Analysis

The statistical analysis was performed using the GraphPad Prism software V10.2.0. The ordinary one-way analysis of variance (ANOVA) with Turkey’s multiple comparisons test was used to analyze the differences between multiple groups of data. * *p* < 0.05 was defined as a statistically significant difference.

## 5. Conclusions

Fungal exopolysaccharides extracted from submerged mycelial culture broth of *S. commune* CMU-01 confer indirect mechanisms against *Salmonella* by (1) reducing *Salmonella* gut epithelial cell invasion and (2) enhancing macrophage function. However, the essential role of *S. commune* EPs in the gut environment of *Salmonella*-infected individuals (or animals) should be further explored.

## Figures and Tables

**Table 1:** Chemical characteristics of the crude EPs of *S*. *commune* CMU-01.

Wavenumber (cm^−1^)	Characteristics and Functional Groups of the Peaks
3253.8 to 3280.4	Stretching vibrations of hydroxyl group (–OH)
2885.9 to 2918.4	C–H stretching vibrations of aliphatic C–H groups
1627.8 to 1634.3	Stretching vibration of carbonyl group (C=O and C=N)
1361.7 to 1367.1	Bending vibration of C–H and stretching vibration of C–C from aliphatic chain
994.4 to 1028.9	Stretching vibration of C–O, C–O–C, C–O–H and glycosidic linkages

## Data Availability

The datasets presented in this study can be found in online repositories. https://doi.org/10.6084/m9.figshare.31969734.

## Supplementary Materials

The following supporting information can be downloaded at: https://www.mdpi.com/article/10.3390/ijms27104476/s1.
