# Supplementary material for: Schizophyllum commune Exopolysaccharides Reduce Salmonella Gut Epithelial Invasion and Activate Macrophages Towards M1-Polarization
Source: Int J Mol Sci. 2026 May 16;27(10):4476. doi: 10.3390/ijms27104476 (PMC13207472; doi:10.3390/ijms27104476)
Supplement: Supplementary file 1 [file ijms-27-04476-s001.zip › ijms-4278560-supplementary.pdf]

SUPPLEMENTARY MATERIALS: (Tantibhadrasapa et al.)

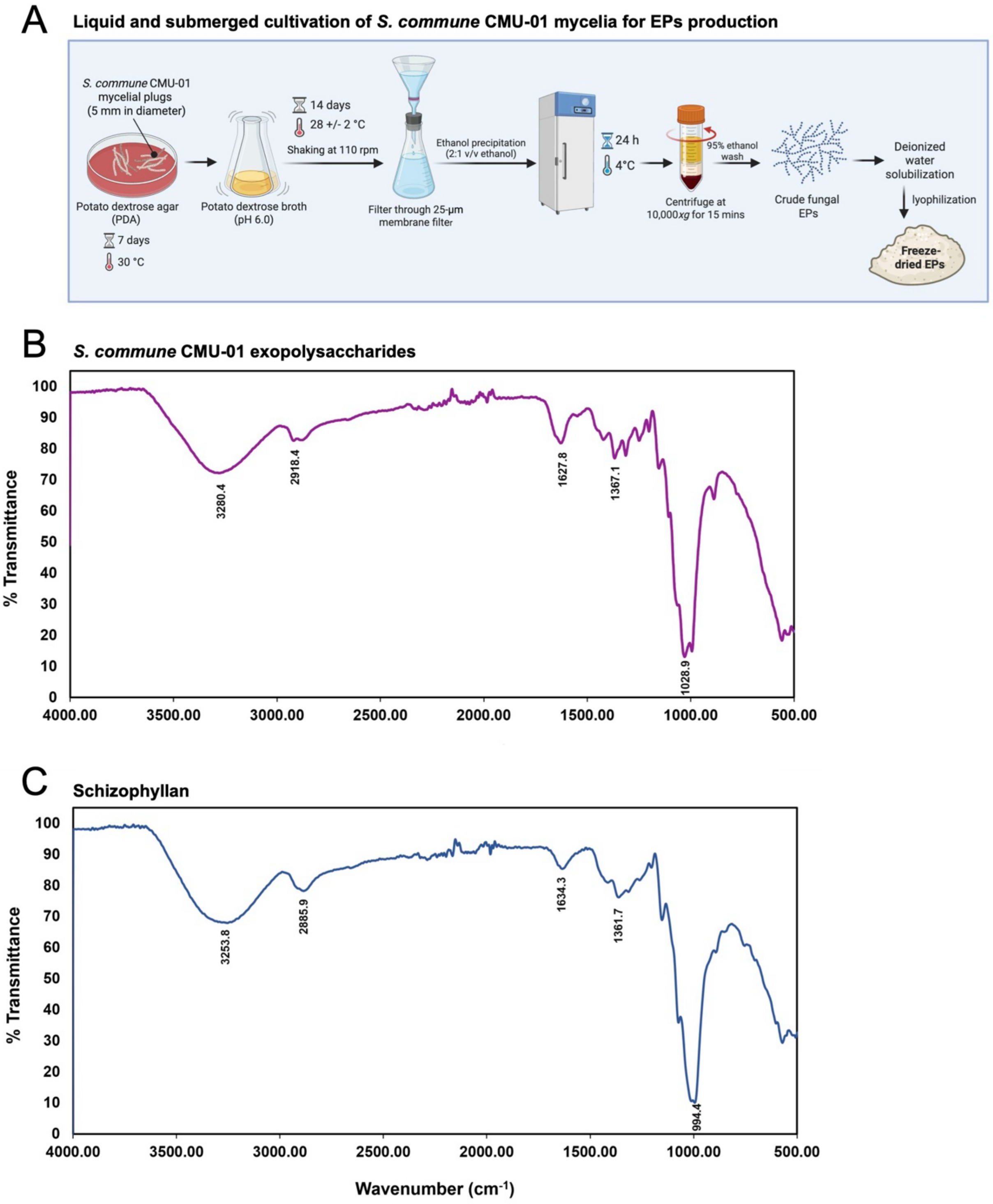

Figure S1. The steps of *S. commune* CMU-01 crude EPs preparation (A). The typical carbohydrate patterns of *S. commune* CMU-01 crude EPs (B) and the commercial schizophyllan (Biosynth®, United Kingdom) (C) were analyzed and compared using FT-IR spectroscopy.

Supplementary Table S1. Bacterial and mushroom strains used in this study

Designation	Genotype and relevant characteristic	Reference
Salmonella enterica Typhimurium IR715	Nalidixic acid resistance derivative of ATCC 14028	(1)
Schizopyllum commune CMU-01	Wild-type strain isolated from natural basidiomata and deposited in the Culture Collection of the Research Center of Microbial Diversity and Sustainable Utilization, Faculty of Science, Chiang Mai University, Thailand	(2)

Supplementary Table S2. The primers used in this study.

Gene		Sequence (5'->3')	Reference
Mouse *Gapdh*	Forward	TGTAGACCATGTAGTTGAGGTCA	(3)
	Reverse	AGGTCGGTGTGAACGGATTTG	
Mouse *Kc*	Forward	TGCACCCAAACCGAAGTCAT	
	Reverse	TTGTCAGAAGCCAGCGTTCAC	
Mouse *Nos2*	Forward	CCAGCCTTGCATCCTCATTGG	
	Reverse	CCAAACACCAAGCTCATGCGG	
Mouse *Tnfa*	Forward	TTGGGTCTTGTTCACTCCACGG	(4)
	Reverse	CCTCTTTCAGGTCACTTTGGTAGG	
Mouse *Mip2*	Forward	AGTGAACTGCGCTGTCAATGC	(5)
	Reverse	AGGCAAACTTTTTGACCGCC	
Mouse *Il6*	Forward	GCACAACTCTTTTCTCATTTCCACG	(6)
	Reverse	GCCTTCCCTACTTCACAAGTCCG	
Mouse *Il10*	Forward	GGTTGCCAAGCCTTATCGGA	
	Reverse	ACCTGCTCCACTGCCTTGCT	

Mouse *Tlr2*	Forward	AGCTCTTTGGCTCTTCTG	(7)
	Reverse	AGAACTGGGGGATATGC	
Mouse *Tlr4*	Forward	GCATGGCTTACACCACCTCT	
	Reverse	GTGCTGAAAATCCAGGTGCT	
Mouse *Cd11c*	Forward	GTGCCCATCAGTTCCTTACA	(8)
	Reverse	GAGAAGAACTGTGGAGCTGAC	
Mouse *Cd206*	Forward	GGAATCAAGGGCACAGAGTTA	
	Reverse	ATTGTGGAGCAGATGGAA	
Human *GAPDH*	Forward	CCAGGAAATGAGCTTGACAAAGT	(9)
	Reverse	CCCACTCCTCCACCTTTGAC	
Human *IL8*	Forward	GCCAACACAGAAATTATTGTAAAGCTT	(10)
	Reverse	CCTCTGCACCCAGTTTTCCTT	
Human *MIP3A*	Forward	CTGCTTTGATGTCAGTGCTGCTAC	(11)
	Reverse	CTGCCGTGTGAAGCCCACAATAAA	
Human *IL1B*	Forward	AAACAGATGAAGTGCTCCTTCCAGG	(12)
	Reverse	TGGAGAACACCACTTGTTGCTCCA	
Human *TNFa*	Forward	CGGGACGTGGAGCTGGCCGAGGAG	
	Reverse	CACCAGCTGGTTATCTCTCAGCTC	
Human *IL10*	Forward	ATGCCCCAAGCTGAGAACCAAGACCCA	
	Reverse	TCTCAAGGGGCTGGGTCAGCTATCCCA	
Human *DECTIN1*	Forward	CTGGGAGGATGGATCAACAT	(13)
	Reverse	CTGACACGTGAATCCATACACA	

REFERENCES

1. Stojiljkovic I, Baumler AJ, Heffron F. Ethanolamine utilization in Salmonella typhimurium: nucleotide sequence, protein expression, and mutational analysis of the cchA cchB eutE eutJ eutG eutH gene cluster. J Bacteriol. 1995;177(5):1357-66.
2. Chotmanee T, Suwannarach N, Kumla J, Phongphisutthinant R, Chaipoot S, Wiriyacharee P, et al. Exopolysaccharide production by seven basidiomycetous fungi and their antioxidant and immunomodulatory activities against Salmonella infection. Front Cell Infect Microbiol. 2025;15:1610403.
3. Winter SE, Thiennimitr P, Winter MG, Butler BP, Huseby DL, Crawford RW, et al. Gut inflammation provides a respiratory electron acceptor for Salmonella. Nature. 2010;467(7314):426-9.
4. Winter SE, Winter MG, Xavier MN, Thiennimitr P, Poon V, Keestra AM, et al. Host-derived nitrate boosts growth of E. coli in the inflamed gut. Science (New York, NY). 2013;339(6120):708-11.
5. Winter SE, Winter MG, Thiennimitr P, Gerriets VA, Nuccio SP, Russmann H, et al. The TviA auxiliary protein renders the Salmonella enterica serotype Typhi RcsB regulon responsive to changes in osmolarity. Molecular microbiology. 2009;74(1):175-93.
6. Xavier MN, Winter MG, Spees AM, Nguyen K, Atluri VL, Silva TM, et al. CD4+ T cell-derived IL-10 promotes Brucella abortus persistence via modulation of macrophage function. PLoS pathogens. 2013;9(6):e1003454.
7. Applequist SE, Wallin RP, Ljunggren HG. Variable expression of Toll-like receptor in murine innate and adaptive immune cell lines. Int Immunol. 2002;14(9):1065-74.
8. Zhu Y, Zhang L, Lu Q, Gao Y, Cai Y, Sui A, et al. Identification of different macrophage subpopulations with distinct activities in a mouse model of oxygen-induced retinopathy. Int J Mol Med. 2017;40(2):281-92.
9. Raffatellu M, Chessa D, Wilson RP, Dusold R, Rubino S, Baumler AJ. The Vi capsular antigen of Salmonella enterica serotype Typhi reduces Toll-like receptor-dependent interleukin-8 expression in the intestinal mucosa. Infection and immunity. 2005;73(6):3367-74.
10. Stylianou E, Yndestad A, Sikkeland LI, Bjerkeli V, Damas JK, Haug T, et al. Effects of interferon-alpha on gene expression of chemokines and members of the tumour necrosis factor superfamily in HIV-infected patients. Clin Exp Immunol. 2002;130(2):279-85.
11. Johanesen PA, Dwinell MB. Flagellin-independent regulation of chemokine host defense in Campylobacter jejuni-infected intestinal epithelium. Infection and immunity. 2006;74(6):3437-47.
12. Jung HC, Eckmann L, Yang SK, Panja A, Fierer J, Morzycka-Wroblewska E, et al. A distinct array of proinflammatory cytokines is expressed in human colon epithelial cells in response to bacterial invasion. J Clin Invest. 1995;95(1):55-65.
13. Gong J, Wang P, Qiu ZH, Chen QJ. Increased expression of dectin-1 in nasal polyps. Am J Otolaryngol. 2013;34(3):183-7.
